# An enantiomerically pure siderophore type ligand for the diastereoselective 1 : 1 complexation of lanthanide(III) ions

**DOI:** 10.3762/bjoc.5.78

**Published:** 2009-12-11

**Authors:** Markus Albrecht, Olga Osetska, Thomas Abel, Gebhard Haberhauer, Eva Ziegler

**Affiliations:** 1 Institut für Organische Chemie, RWTH Aachen University, Landoltweg 1, 52074 Aachen, Germany; 2 Institut für Organische Chemie, Universität Duisburg-Essen, Universitätsstraße 7, 45117 Essen, Germany

**Keywords:** CD spectroscopy, enterobactin, lanthanide, macrocycles, molecular modeling

## Abstract

A facile synthesis of a highly preorganized tripodal enterobactine-type ligand **1a**-H_3_ consisting of a chiral *C*_3_-symmetric macrocyclic peptide and three tridentate 2-amido-8-hydroxyquinoline coordinating units is presented. Complex formation with various metal ions (Al^3+^, Ga^3+^, Fe^3+^, La^3+^ and Eu^3+^) was investigated by spectrophotometric methods. Only in the case of La^3+^ and Eu^3+^ were well defined 1 : 1 complexes formed. On the basis of CD spectroscopy and DFT calculations the configuration at the metal centre of the La^3+^ complex was determined to show *Λ* helicity. The coordination compounds [(**1a**)Ln] presented should be prototypes for further lanthanide(III) complexes with an enterobactine analogue binding situation.

## Introduction

The availability of metal ions for biological systems is essential for growth and function. Therefore microorganisms have to develop strategies how to solubilise and take up charged ions through highly non-polar membranes [1–2].

**Figure 1:**
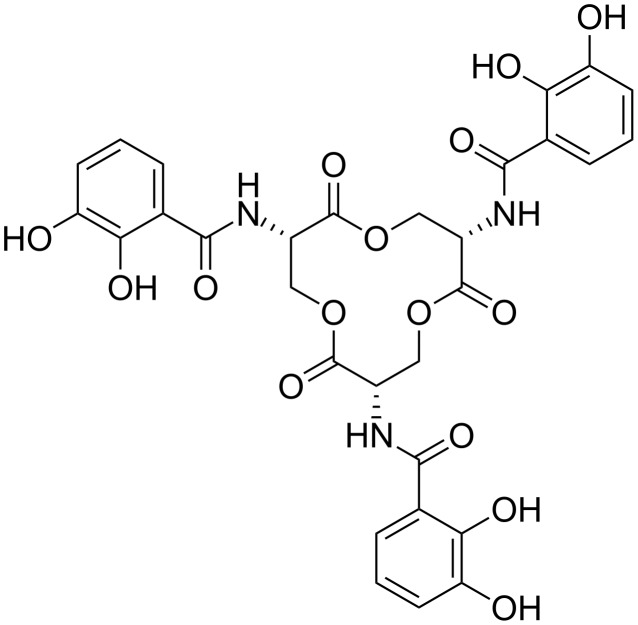
Structural formula of the siderophore enterobactine.

An important class of natural products which is responsible for the uptake of iron are the siderophores [3–5]. The probably most prominent example of this class is enterobactin (Figure 1) [6–8]. It has inspired the synthesis of a wide series of non-natural compounds which are used for metal ion binding and medical purposes [9–22]. However, the efficiency of this artificial chelators to bind iron(III) excels only in few cases the one observed for enterobactin [23–24].

Enterobactin resembles the ideal raw model for the design of highly efficient metal ion receptors. It combines two different structural aspects which are important for effective binding of the metals: (i) **Chelating moieties**: Catechol is an efficient chelating moiety, which can form highly stable complexes with a series of first row transition metals. (ii) **Chirality**: The enterobactin-backbone is based on L-serine units and therefore is chiral (and enantiomerically pure). This chiral information gives all three ligand units the same spatial orientation, leading to a bowl-shape structure which is preorganized for the uptake of the metal. Here we present the synthesis of a novel tripodal ligand, which mimics some features of enterobactin, but in contrast is specific for the binding of high coordinated lanthanide(III) ions. The backbone is based on an enantiomerically pure “non-natural” peptide moiety [25], which organizes three tridenate metal binding sites in one direction [26–28]. The synthesis of the compound is facile and utilizes a multiple Claisen-rearrangement reaction as introduced by Hiratani as the keystep [29–30]. UV and CD titration experiments use the constricted chirality in the metal complex and allow the investigation of the binding of f-element cations and the determination of the selectivity towards this class of metal ions.

## Results and Discussion

### Synthesis of the Ligand

**Scheme 1:**
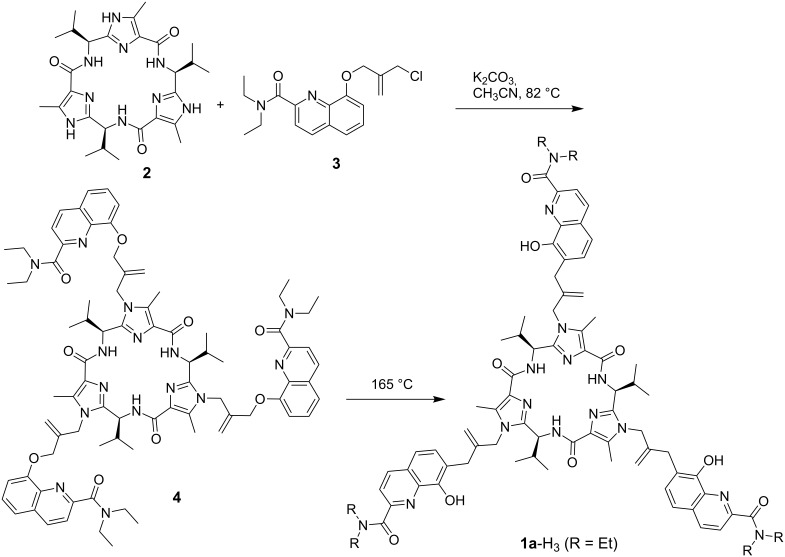
Preparation of the compound **1a**-H_3_ by utilization of a multiple Claisen-rearrangement.

The preparations of the required building blocks **2** [31–32] and **3** [33] for the synthesis of the ligand **1a**-H_3_ have already been described before (Scheme 1).

The *C*_3_-symmetric scaffold **2** is obtained in an eight step sequence starting from Z-protected valine and methyl 2-amino-3-oxobutanoate hydrochloride [31–32]. Compound **2** resembles the ideal platform for an enterobactine-type ligand system. It possesses a concave shape in which the three anchor points at the NH units of imidazole are orientated towards the same direction in space. The ether **3** is attached to this position by an S_N_-reaction. Derivative **3** possesses a masked 8-hydroxyquinoline unit which is extended to be tridentate by addition of diethylamide to the 2-position. Recently 2-amido-8-hydoxyquinolines were shown to be good ligands for the 3 : 1 complexation of lanthanide(III)ions [34–35]. The aryl ether of **3** bears already the allylic unit for Claisen rearrangement as well as the chloride leaving group for the attachment of other units. Coupling of **2** with three equivalents of **3** results in the formation of the ligand precursor **4**. The triple Claisen rearrangement of **4** proceeds at 165 °C under inert atmosphere (N_2_) within 6 hours [36]. No epimerization occurs at the chiral carbon atoms of the peptidic scaffold and the final ligand **1a**-H_3_ is obtained in good yield (90%). Successive Cope rearrangement of the spacers to the 5-position of the quinoline moiety is not observed under the chosen reaction conditions.

**Figure 2:**
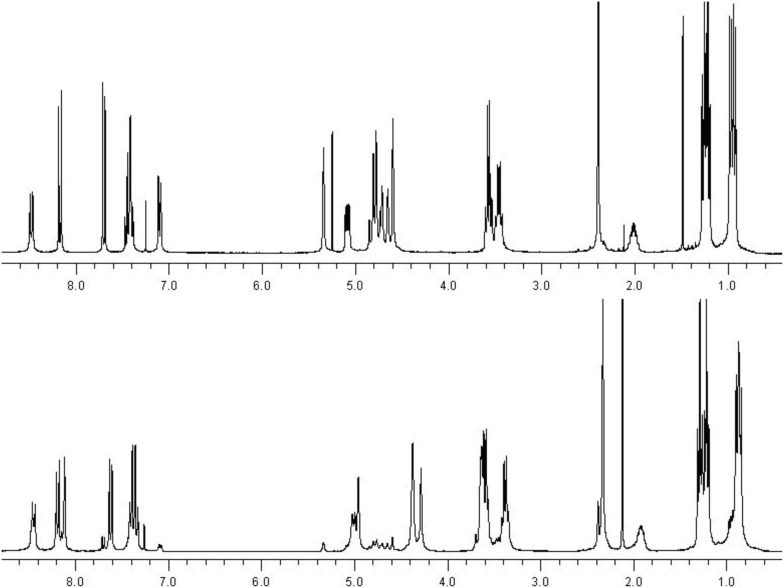
^1^H NMR spectra (300 MHz, CDCl_3_) of the ether compound **4** (top) and the ligand **1a**-H_3_ (bottom).

^1^H NMR spectra of **4** and **1a**-H_3_ show pronounced differences for the resonances of the 8-hydroxyquinoline moiety and the spacer, while the signals of the backbone do not change significantly (Figure 2). Most remarkable is the disappearance of the signal of the proton in 7-position of **4** at δ = 7.06 ppm. The vinylic hydrogen atoms appear for the precursor **4** at δ = 5.29 and 4.55 ppm and are shifted in **1a**-H_3_ to δ = 4.91 and 4.24 ppm. Differences are also observed for the CH_2_ units of the spacer. In **4** they are both observed as multiplets around δ = 4.71 ppm, while they appear separated in **1a**-H_3_ as a singlet at δ = 4.33 ppm (N–CH_2_) and as a signal which is hidden under the methylene units of the diethylamide at δ = 3.56 ppm (C_aryl_–CH_2_).

### Coordination studies

**Figure 3:**
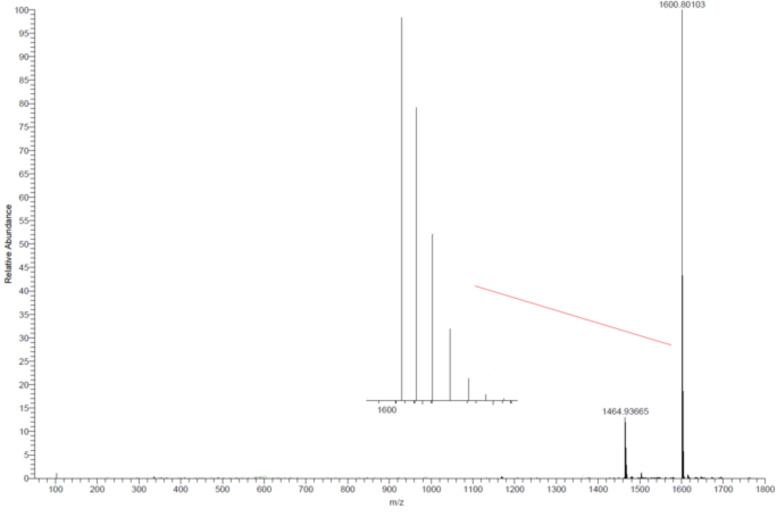
Positive ESI MS of [(**1a**)La] in chloroform showing the peaks of {K[(**1a**)La]}^+^ (m/z = 1600.8) as well as of {K(**1a**-H_3_)}^+^ (m/z = 1464.9). The inset shows the isotopic pattern of the peak at m/z = 1600.8, which corresponds to the one calculated for {K[(**1a**)La]}^+^.

In an orientating coordination study we reacted the ligand **1a**-H_3_ on a preparative scale (30 mg) with lanthanum(III) chloride heptahydrate in the presence of potassium carbonate as base in methanol at room temperature. After four days solvent was distilled off and the residue was washed with water in order to remove potassium chloride and uncoordinated lanthanide salts. The complex was obtained in 82% yield as a red solid with elemental analysis correct for the pentahydrate of [(**1a**)La]. NMR spectroscopy of the complex did not show significant shift differences between the ligand and the complex. This could be due to the lability of the compound in solution and fast dissociation/association equilibria. However, positive ESI MS in chloroform showed the base peak at m/z = 1600.8 for {K[(**1a**)La]}^+^ with correct isotopic pattern (Figure 3). Due to the already described dissociation equilibrium in solution uncoordinated ligand can be observed as well at m/z = 1464.9 {K(**1a**-H_3_)}^+^.

### UV and CD titration experiments

**Figure 4:**
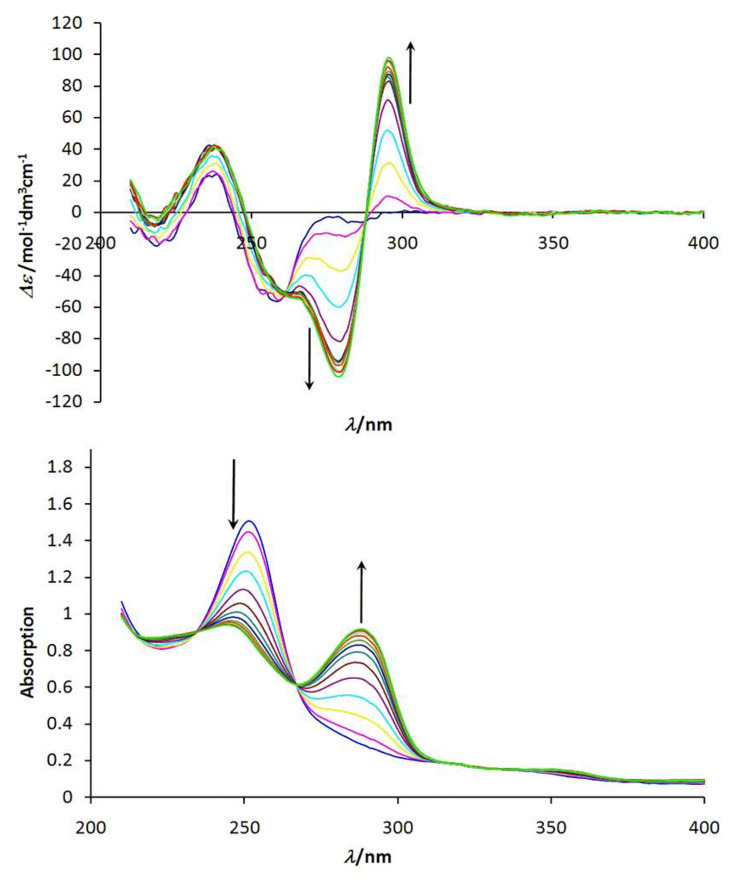
CD and UV absorption titration curves for complexation of ligand **1a**-H_3_ with lanthanum(III)nitrate hexahydrate [**1a**-H_3_] = 10^−5^M; 10^−4^ M NaOH. Top: CD spectra; bottom: UV absorption spectra.

As a sensitive technique for the investigation of the complex formation of ligand **1a**-H_3_ with a series of trivalent metal ions we performed UV–vis as well as CD spectroscopic titrations in methanol together with NaOH as base (10^−4^ M; Figure 4). Upon coordination of the metal ions to the ligand the transitions at the aromatic unit are influenced leading to changes in the UV–vis spectrum. In addition, metal coordination restricts the conformation at the ligand leading to a significant change of the observed CD spectra.

Small trivalent metal ions like aluminium(III), gallium(III) or iron(III) which are able to form (distorted) octahedral coordination compounds lead to UV–vis spectra which show isosbestic behaviour. However, the titration curves show more or less linear changes of the absorption (in case of iron(III) a kink is observed at 1.5–2.0 equivalents iron(III) salt added). This indicates that not a 1 : 1 but probably polymeric coordination compounds are formed.

**Figure 5:**
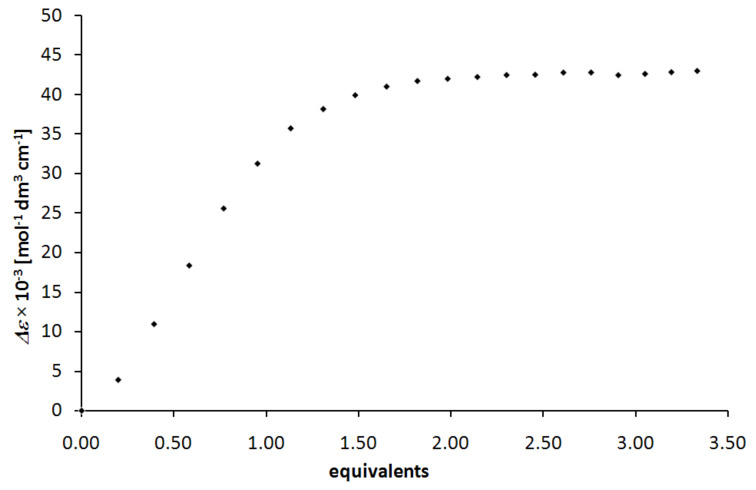
Titration curve observed for ligand **1a**-H_3_ upon addition of lanthanum(III) nitrate hexahydrate.

Only titrations with lanthanum(III) or europium(III) salts show well defined reliable behaviour (Figure 5). The spectra for the two different metal ions are very similar indicating that the observed transitions are ligand-centered. CD as well as UV spectra of the titration of **1a**-H_3_ with lanthanum(III) are shown in Figure 4, while Figure 5 depicts the corresponding titration curve following the absorption at 279 nm.

Analysis of the titration data reveals high binding constants for lanthanum(III) (*K*_a_ = 8.3 × 10^5^ M^−1^) as well as europium(III) (*K*_a_ = 7.8 × 10^5^ M^−1^) for the reaction of deprotonated ligand **1a**^3−^ with the Ln^3+^ ion to form [(**1a**)Ln] in methanol at room temperature.

### Ab initio calculations

In order to determine the configuration at the metal centres of the **1a·**Ln (R = Et) complexes ab initio calculations were performed for the stereoisomers of complex **1b·**La (R = Me) [37]. The difference between **1a** and **1b** is only the substitution of the ethyl groups in **1a** by methyl groups in **1b**. This simplification reduces the number of optimization steps in the calculations and should not affect the extent of diastereoselectivity for the complex formation. In principle the *C*_3_-symmetric **1b·**La complex can adopt four different conformations (*Λ1*, *Λ2*, *Δ1*, *Δ2*): The 8-hydroxyquinoline units can be present in two opposite helicities (*Λ* and *Δ*) and the three isobutenylidene spacers can adopt two different orientations relative to the macrocycle: the CH_2_ units of the spacers point to the interior of the molecule in the case of the conformers (*Λ1*)-**1b·**La and (*Δ1*)-**1b·**La and to the exterior in the case of the conformers (*Λ2*)-**1b·**La and (*Δ2*)-**1b·**La. The structures of the complex **1b·**La were determined by geometry optimizations at DFT-level by using B3LYP/LANL2DZ.

**Figure 6:**
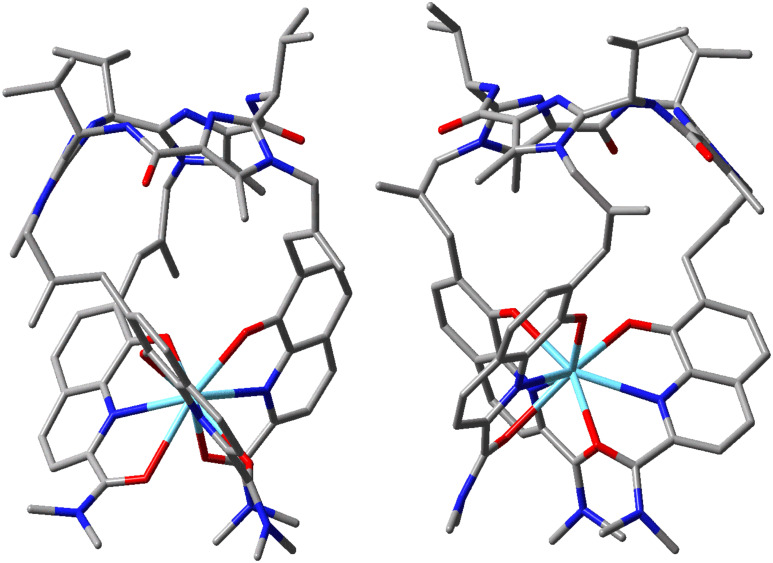
Molecular structures of the *Λ2* (left) and *Δ2* (right) isomers of complex **1b·**La calculated by using B3LYP/LANL2DZ. All hydrogen atoms were omitted for clarity.

The calculations revealed that the *Λ2* isomer is the energetically favored one (Figure 6). The energies of the other conformers were calculated to be much higher (38 kJ mol^−1^ for *Δ2*, 49 kJ mol^−1^ for *Λ1* and 52 kJ mol^−1^ for *Δ1*) relative to the *Λ2* isomer. On the basis of these high energy differences between the four diastereomers and the assumption that the enthalpy of hydrolysis does not differ significantly for the four possible helical isomers, the *Λ2* isomer should be the only *C*_3_-symmetric isomer present in solution.

**Figure 7:**
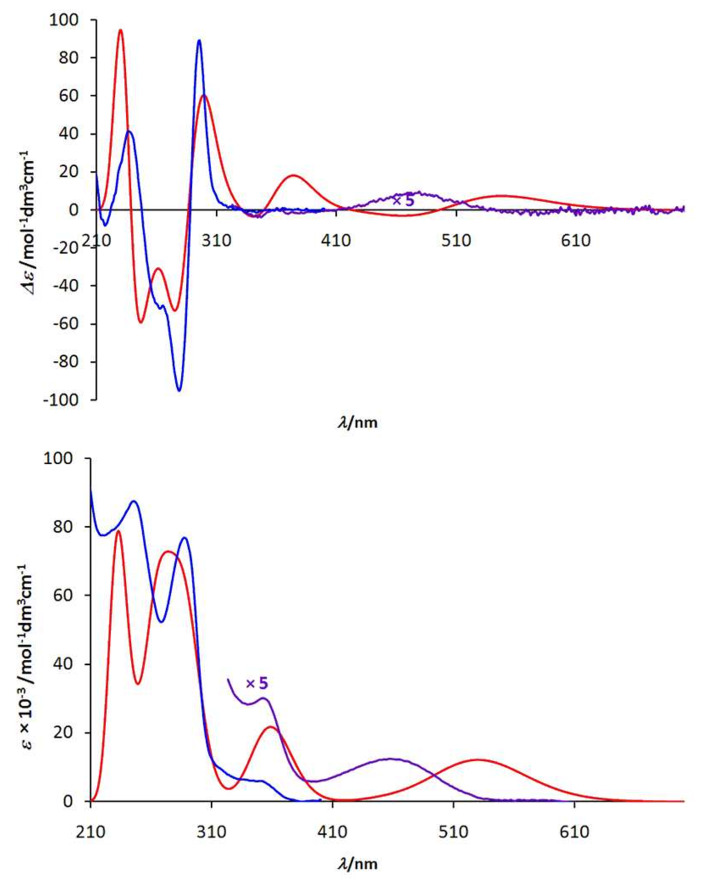
UV and CD spectra of the complex (*Λ*)-**1·**La. Blue and violet curve: experimentally determined spectra of (*Λ*)-**1·**La; red curve: calculated spectrum of the *Λ2* isomer of complex **1b·**La calculated at the TD-DFT-PBE1PBE/LANL2DZ level.

As further evidence that the formation of the **1·**La complex is strictly diastereoselective the UV and CD spectra of the (*Λ2*)-**1b·**La complex were simulated on the basis of time-dependent density functional theory (TD-DFT) with the PBE1PBE functional and by employing the LANL2DZ basis set [37]. TD-DFT calculations were performed at the optimized ground-state geometry (B3LYP/LANL2DZ), calculating the energy, oscillator strength and rotatory strength for each of the 200 lowest singlet excitations. The CD spectrum was simulated by overlapping Gaussian functions for each transition where the width of the band at 1/*e* height was fixed at 0.4 eV and the resulting intensity of the combined spectrum was scaled to the experimental values (Figure 7).

A comparison of the calculated spectrum with the experimentally determined one shows that the complex exhibits *Λ2* conformation in solution, too. The positive Cotton effect at 295 nm as well as the negative Cotton effect at 280 nm are found in both spectra. These effects derive from an exciton coupling [38] of the 8-hydroxyquinoline chromophore and therefore they can be used for the unambiguous determination of the helicity. Even the Cotton effects at around 350 nm are of the same sign in both spectra. However, the calculations overestimate the intensity of this excitation; this overestimation can also be found in the calculated UV spectrum. Moreover, in both spectra the excitations at higher wavelengths exhibit a positive Cotton effect. As the calculated maximum of the UV spectrum (530 nm) shows a bathochromic shift relative to the measured UV spectrum (470 nm), the corresponding peak in the calculated CD spectrum (540 nm) is shifted to higher wavelengths relative to the experimentally measured one (480 nm).

## Conclusion

We present the facile synthesis of a highly preorganized tripodal enterobactine-type ligand **1a**-H_3_ consisting of the chiral *C*_3_-symmetric backbone **2** and three tridentate 2-amido-8-hydroxyquinoline coordinating units. The ligand units can be easily attached to the backbone by an alkylation reaction followed by a triple Claisen rearrangement as described by Hiratani. In this way three new C–C bonds are formed in one reaction step and three isobutenylidene spacers are installed.

UV–vis and CD titrations show that ligand **1a**^3−^ forms well defined 1:1 complexes only with lanthanide(III) ions, while smaller cations lead to an unspecific complex formation (probably oligomerization or polymerization). On the basis of ab initio calculations and CD spectroscopy we could show that the formed complex (**1a**)La exhibits exclusively *Λ* helicity.

We were able to isolate the corresponding lanthanum(III) complex as solid material and to characterize it by elemental analysis and positive ESI MS.

The coordination compounds [(**1a**)Ln] we describe should be prototypes for further lanthanide(III) complexes with an enterobactine analogue binding situation. In this context the strong complexation of lanthanide(III) ions is of interest, due to the special properties of those compounds as light emitting or magnetic materials [39].

## Experimental

NMR spectra were recorded on a Varian Mercury 300 NMR spectrometer. FT-IR spectra were measured on a PerkinElmer Spektrum 100 spectrometer. Mass spectra were taken on a ThermoFisher Scientific LTQ-Orbitrap XL mass spectrometer. Elemental analyses were obtained with a Heraeus Elementar Vario EL analyser.

### Synthesis and characterization of macrocyclic imidazole peptide **4**

To **2** (80 mg, 0.15 mmol) in acetonitrile (80 ml) were added anhydrous potassium carbonate (400 mg, 1.20 mmol) and **3** (200 mg, 0.62 mmol). The solution was heated and refluxed for 12 h. After cooling to room temperature the solution was stirred 12 h and afterwards concentrated to dryness. The residue was dissolved in ethyl acetate, washed with water and brine and dried with anhydrous magnesium sulfate. The solution was concentrated to dryness. The crude product was purified by column chromatography (silica gel; methylene chloride/ethyl acetate/methanol, 75:25:10; R_f_ = 0.37) to yield **4** as a brown solid.

Yield: 81 mg (41 %); mp 107–108 °C. ^1^H NMR (300 MHz, CDCl_3_): δ = 8.43 (d, *J* = 8.9 Hz, 3H), 8.13 (d, *J* = 8.9 Hz, 3H), 7.65 (d, *J* = 8.4 Hz, 3H), 7.38 (m, 6H), 7.06 (dd, *J* = 1.6/7.8 Hz, 3H), 5.29 (s, 3H), 5.04 (q, *J* = 4.6 Hz, 3H), 4.71 (m, 12H), 4.55 (s, 3H), 3.53 (q, *J* = 7.2 Hz, 6H), 3.42 (q, *J* = 7.2 Hz, 6H), 2.35 (s, 9H), 1.97 (m, 3H), 1.21 (t, *J* = 7.2 Hz, 9H), 1.17 (t, *J* = 7.2 Hz, 9H), 0.93 (d, *J* = 6.68 Hz, 9H), 0.89 (d, *J* = 6.68 Hz, 9H). IR (KBr): 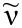 (cm^−1^) = 3383 (m), 3062 (w), 2965 (m), 2933 (m), 2873 (m), 2360 (w), 1658 (vs), 1633 (vs), 1595 (s), 1462 (s), 1423 (s), 1377 (s), 1325 (m), 1254 (m), 1205 (m), 1112 (s), 990 (w), 922 (w), 846 (m), 763 (m), 633 (w). HRMS (ESI): calcd. for C_81_H_99_N_15_O_9_ [M + Na]^+^: m/z = 1448.7642; found 1448.7639. HRMS (ESI): calcd. for C_81_H_99_N_15_O_9_ [M + H]^+^: m/z = 1426.7823; found 1426.7808. HRMS (ESI): [M + 2Na]^++^: m/z = 735.8739; found 735.8767.

### Synthesis and characterization of macrocyclic imidazole peptide based tris(*N*,*N*-diethyl-8-hydroxyquinoline-2-carboxamide) **1a**-H**_3_**

The ether **4** (0.18 g, 0.13 mmol) was placed in a Schlenk flask. The rearrangement proceeded at 165 °C under dry inert atmosphere of N_2_. The dark brown residue was purified by a short silica column (EtOAc, R_f_ = 0.26) to furnish a bright brown solid.

Yield: 0.16 g (90 %); mp 189–196 °C. ^1^H NMR (300 MHz, CDCl_3_): δ = 8.40 (d, *J* = 8.7 Hz, 3H), 8.14 (d, *J* = 8.7 Hz, 3H), 8.07 (s, 3H), 7.57 (d, *J* = 8.4 Hz, 3H), 7.34 (m, 6H), 4.96 (q, *J* = 4.3 Hz, 3H), 4.91 (s, 3H), 4.33 (s, 6H), 4.24 (s, 3H), 3.56 (m, 12H), 3.33 (q, *J* = 7.0 Hz, 6H), 2.28 (s, 9H), 1.87 (m, 3H), 1.24 (t, *J* = 7.0 Hz, 9H), 1.17 (t, *J* = 7.0 Hz, 9H), 0.84 (d, *J* = 6.8 Hz, 9H), 0.81 (d, *J* = 6.8 Hz, 9H). IR (KBr): 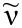 (cm^−1^) = 3383 (m), 3083 (w), 2964 (m), 2927 (m), 2720 (w), 2283 (w), 2085 (w), 1732 (w), 1629 (vs), 1595 (vs), 1508 (vs), 1453 (vs), 1378 (m), 1321 (m), 1289 (w), 1253 (m), 1204 (s), 1139 (w), 1107 (s), 1021 (m), 992 (w), 909 (m), 848 (s), 809 (w), 774 (m), 723 (m), 662 (w). HRMS (ESI): calcd. for C_81_H_99_N_15_O_9_ [M + Na]^+^: m/z = 1448.7619; found 1448.7637.

### Synthesis and characterization of a mononuclear lanthanum(III) complex with cyclohexapeptide based tris(*N*,*N*-diethyl-8-hydroxyquinoline-2-carboxamide) [(**1a**)La]

LaCl_3_ · 7 H_2_O (0.008g, 0.02 mmol, 1.0 equiv.) and K_2_CO_3_ (0.009 g, 0.06 mmol, 3.0 equiv.) in MeOH / H_2_O (4 ml / 1 ml) were added to ligand **2** (0.030 g, 0.02 mmol, 1.0 equiv.) in MeOH (10 ml). The mixture was stirred at RT for 4 days. The solution was concentrated under reduced pressure and the red coloured residue was washed with water.

Yield: 0.027 g (82%); mp 242–248 °C (dec.). Positive ESI MS (chloroform): m/z (%) = 1600.8 ([C_81_H_96_N_15_O_9_LaK]^+^, 100), 1464.9 ([C_81_H_99_N_15_O_9_K]^+^, 14). C_81_H_96_N_15_O_9_La · 5 H_2_O: C 58.87, H 6.46, N 12.71; found: C 58.38, H 6.62, N 12.62.

To determine the thermodynamic parameters of the metal complexes all titration experiments were accomplished at room temperature by using a Jasco J-815 spectrophotometer connected to an automatic titration unit (Jasco ATS-443). For this purpose a methanolic solution containing ligand **1a**-H_3_ (10^−5^ M) with NaOH (10^−4^ M) and a titrant solution containing the metal salt ([**1a**-H_3_] = 10^−5^ M, [M^3+^] = 2 × 10^−4^ M, [NaOH] = 10^−4^ M in MeOH) were prepared. The titrant solution was added in discrete steps to the solution containing ligand **1a**-H_3_. After a mixing time of 2 min the spectra were recorded.

The virtual binding constants were evaluated according to Equation 1. It represents a simple association constant which involves all protonation/deprotonation and metal ion coordination steps.

[1]
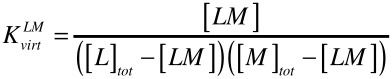


The virtual association constants of the complexation systems were calculated by non-linear-square fitting according to the Benesi–Hildebrand equation from the UV absorbtion data set. For a simple 1 : 1 binding model the calculations were carried out with the SigmaPlot program. Best results and lowest error could be observed at 279 nm in the case of lanthanum(III) and at 281 nm in the case of europium(III).
